# AUF1 Promotes Proliferation and Invasion of Thyroid Cancer *via* Downregulation of ZBTB2 and Subsequent TRIM58

**DOI:** 10.3389/fonc.2021.681736

**Published:** 2021-06-16

**Authors:** Xin Du, Jia-Mei Wang, Da-Lin Zhang, Tong Wu, Xiao-Yan Zeng, Jing-Yi Jiang, Zhen-Xian Du

**Affiliations:** ^1^ Department of Endocrinology and Metabolism, The First Affiliated Hospital of China Medical University, Shenyang, China; ^2^ Department of Laboratory Medicine, The First Affiliated hospital of China Medical University, Shenyang, China; ^3^ Department of Thyroid Surgery, The First Affiliated Hospital of China Medical University, Shenyang, China; ^4^ Department of Biochemistry and Molecular Biology, China Medical University, Shenyang, China

**Keywords:** AUF1, ZBTB2, TRIM58, papillary thyroid cancer, proliferation and invasion

## Abstract

The pathogenesis of papillary thyroid cancer (PTC), the most common type of thyroid cancer, is not yet fully understood. This limits the therapeutic options for approximately 7% of invasive PTC patients. The critical role of AUF1 in the progression of thyroid cancer was first reported in 2009, however, its molecular mechanism remained unclear. Our study used CRISPR/Cas 9 system to knockdown AUF1 in IHH4 and TPC1 cells. We noticed that the expression of TRIM58 and ZBTB2 were increased in the AUF1 knockdown IHH4 and TPC1 cells. When TRIM58 and ZBTB2 were inhibited by small hairpin RNAs (shRNAs) against TRIM58 (shTRIM58) and ZBTB2 (shZBTB2), respectively, the proliferation, migration, and invasion ability of the AUF1-knockdown IHH4 and TPC1 cells were increased. In addition, two ZBTB2 binding sites (-719~-709 and -677~-668) on TRIM58 promoter and two AUF1 binding sites (1250-1256 and 1258-1265) on ZBTB2 3’-UTR were identified. These results suggested that AUF1 affecting thyroid cancer cells *via* regulating the expression of ZBTB2 and TRIM58.

## Introduction

Thyroid cancer is one of the most common endocrinal malignancies, which is predicted to become the fourth most prevalent cancer worldwide by 2030 (1–4). Thyroid cancer can be classified into four major types, namely papillary thyroid carcinoma (PTC), follicular thyroid carcinoma, anaplastic thyroid carcinoma, and medullary thyroid carcinoma (5). Among which, PTC accounts for 80–85% of all thyroid cancers (6, 7). Although PTC has an excellent 5-year survival rate (8), there are still about 7% of patients with invasive PTC that lack effective therapeutic options. Therefore, there is a dire need of understanding PTC’s tumorigenesis and seeking novel treatment approaches.

The stability of mRNA and its correct translation is important basis for cells to respond to external stimuli (9, 10). Stable mRNA is involved in regulating proto-oncogenes to ensure their proper expression, but abnormal regulation can lead to cancer (11). AU-rich RNA-binding factor (AUF1)/heterogeneous nuclear ribonucleoprotein D (hnRNPD) is an adenylate-uridylate (AU)-rich element (ARE)-binding protein, which is located on chromosome 4 (4q21) (12), and regulates the mRNA stability of many genes *via* ARE binding motif with their 3’ untranslated region (UTR) (13, 14). Recent studies revealed that AUF1 promotes the proliferation and invasion ability of breast cancer cells (15), and was closely related to the size and malignancy of hepatic cancer, which might lead to a poorer prognosis (16). Moreover, genetically modified mice that expressed AUF1 were more likely to develop undifferentiated sarcoma (17). A connection between AUF1 and thyroid cancer was first reported in 2009. The study concluded that AUF1 might be used as an additional biomarker for thyroid cancer to distinguish tumors malignancy (18). However, the molecular mechanisms of this effect have not yet been revealed.

Zinc finger and BTB domain 2 (ZBTB2) belongs to the POK transcription factor family, which are involved in transcription, and have been found to be associated with the occurrence of multiple tumors (19–23). TRIM58 is one member of tripartite motif-containing (TRIM) family, the largest families of the E3 ubiquitin connective enzyme, implicated in regulation of protein stability for almost the entire proteome. Many studies revealed that TRIM58 could act as a tumor suppressor. Aberrant gene methylation of TRIM58 was reported in liver and lung cancer and indicates a poor patient prognosis (24, 25). The expression of TRIM58 in the human colorectal cancer was significantly inhibited and negatively correlated with the severity of colorectal cancer (26). In addition, the overexpression of TRIM58 significantly inhibited the growth and tumor causing nature of OS cells of osteoma (27).

In this study, we investigated the effect of AUF1 on proliferation, migration, and invasion of the IHH4 and TPC1 cells. In addition, the molecular mechanisms underlying regulation of ZBTB2 and TRIM58 expression by AUF1 were also explored.

## Materials and Methods

### Cell Culture

IHH-4 cell lines were cultured under standard conditions at 37°C, 5% CO2 atmosphere, and saturated humidity in RPMI-1640 and DMEM medium (Sigma-Aldrich) supplemented with 10% fetal bovine serum. Cells were maintained in logarithmic phase of growth. TPC-1 cell lines were cultured under standard conditions at 37°C, 5% CO2 atmosphere, and saturated humidity in DMEM medium (Sigma-Aldrich) supplemented with 10% fetal bovine serum. Cells were maintained in logarithmic phase of growth. The IHH4 cell lines was obtained from the Health Science Research Resources Bank (Osaka, Japan). The TPC1 cell line was a gift from Professor Meiping Shen (Department of General Surgery, The First Affiliated Hospital of Nanjing Medical University, Nanjing, Jiangsu).

### Lentiviral Vector Construction and Recombinant Lentivirus Production

The lentiviral CRISPR/Cas9-mediated AUF1 gene-editing vectors were generated by annealing gRNA oligonucleotide pairs and subcloning them into a pLenti-Cas9-sgRNA-puro lentivirus vector (GeneChem Co., Ltd.). The shRNA targeting TRIM58 (shTRIM58) or ZBTB2 (shZBTB2) were designed and cloned into a GV118 lentivirus vector (GeneChem Co., Ltd.). DNA sequencing was performed to verify the sequence of the insert, and the identities were 100%. A concentrated lentivirus solution was obtained with a final titer of 1.0 × 10^9^ TU/ml.

### Knockdown of AUF1 by CRISPR/Cas9

Thyroid cancer cells in the logarithmic growth phase were digested with trypsin and inoculated into a six-well plate on the day before cell infection. Packaged sgRNA lentiviral particles were added to infect CAS9 cells to carry out the target cell infection, and incubated at 37°C, 5% CO2 atmosphere. Medium were changed after 10 h of infection. Puromycin was screened after 72 h of infection. Control cells were infected with an empty vector. Identification of knockdown was carried out by real-time RT-PCR and western blot.

### Cell Counting Experiment

Cells in the logarithmic growth phase were seeded in a six-well cell culture plate (1 × 10^5^ cells/well). Each group of cells was divided into three wells, labeled 1–3d respectively, and incubated in saturated humidity incubator at 37°C, 5% CO2 atmosphere. The initial day 0 was set as the baseline of the experiment. Cells in the well labeled 1d were digested and pipette evenly after 24 h of culturing. Cells in wells labeled 2d and 3d were counted in sequence at 48 and 72 h of culturing. The number of cells from each well was respectively counted using a hemocytometer and recorded to perform statistical analysis.

### Colony Formation Assay

TPC1 cells and IHH4 cells were placed in six-well plates, maintained in a medium containing 10% FBS for 14 days. The cells were fixed with 4% paraformaldehyde and stained with 0.4% crystal violet solution for 30 min at room temperature before taken images by an inverted fluorescence microscope.

### Invasion and Migration Assays

TPC1 and IHH4 cells were digested and resuspend in serum-free culture solution before added to the upper chamber of Corning Costar^®^ Transwell 3422 (8.0 μm) culture plates (the Matrigel should be spread 2–4 h in advance for the invasion experiment), while, complete medium containing 10% serum were added to the lower chamber and incubated for 24 h. The cells were fixed with 4% paraformaldehyde for 15 min and stained with 0.1% crystal violet solution for 30 min at room temperature. Inverted fluorescence microscope was performed to take image, and Image J software was used to count migrated cells and perform statistical analysis.

### Western Blot Analysis

Total protein was isolated from cells using cell lysate containing 1% Triton and cocktail of proteinase inhibitors. Protein quantification was analyzed using BCA (Thermo Scientific.23225) method and 20 μg total protein was used for SDS-PAGE electrophoresis. Protein electroporation was transferred to PVDF membrane, and followed by blocking using 5% skimmed milk at room temperature for 1 h. The following antibodies were used: AUF1 (1:1,000 dilution; Cell Signaling Technology, USA), ZBTB2 (1:1,000 dilution; Abcam, UK), TRIM58 (1:1,000 dilution; Abcam, UK), and GAPDH (1:1,000 dilution; Proteintech, USA). After membrane hybridization with secondary antibodies (1:5,000 dilution; Jackson ImmunoResearch, PA, USA) and washing with TBST, protein expression was detected using ECL luminescence. All band intensities were quantified by Image Lab software.

### 5-Ethynyl-2’-Deoxyuridine (Edu) Assay

Cell proliferation was evaluated using Edu staining with Click-iT technology according to the protocol of manufacture (ThermoFisher). Briefly, cells were labeled with 10 mM EdU for 2 h, nuclei were counterstained with blue fluorescent Hoechst 33342. Cells that incorporated Edu were visualized under a fluorescence microscopy.

### Label and Capture Nascent RNA

Newly synthesized RNA was isolated using Click-iT Nascent RNA Capture Kit (Invitrogen, C10365) according to the manufacturer’s instruction. Briefly, cells were incubated in 0.2 mM of 5-ethymyl uridine (EU, an alkyne-modified uridine analog, which is efficiently and naturally incorporated into the nascent RNA) for 4 h and total RNA labeled with EU was isolated using TRIzol reagent (Invitrogen, 15596-018). Then EU-labeled RNA was biotinylated in a Click-iT reaction buffer with 0.5 mM of Biotin azide and subsequently captured on streptavidin magnetic beads.

### RNA Isolation, Reverse Transcription, and Quantitative Polymerase Chain Reaction (qRT-PCR)

Total RNA was isolated from cultures using Trizol reagent (Invitrogen, Carlsbad, CA, USA) according to the manufacturer’s protocol. Reverse Transcription System kit was used to synthesis cDNAs. Quantitative PCR was performed using the SYBR Ex Taq Kit (Takara, Japan) and the expression levels of target genes were normalized against the expression of 18S RNA. For AUF1, the forward and reverse primers were 5’-ACACTAGGACTATGTCGGAG-3’ and 5’-TCGTTCTTACTGGCGTCAATC-3’, respectively. For TRIM58, the forward and reverse primers were 5’-AGTCCTGAGCAGAAGTAAGGC-3’ and 5’-GGATCCAGCTTTACATCCAC-3’, respectively. For ZBTB2, the forward and reverse primers were 5’-AGCTGCAGCACATCTCTGATTC-3’ and 5’-TGTCACAAGTGCTGCACTTG-3’, respectively. For 18S RNA, the forward primer was 5’-CGGACAGGATTGACAGATTGATAGC-3’ and reverse was 5’-TGCCAGAGTCTCGTTCGTTATCG-3’. PCR amplification program: 95°C for 4 min, 94°C for 45 s, 56°C for 45 s, 72°C for 17 min, 25 cycles, 72°C for 10 min. The results of gene amplification were standardized with GAPDH.

### Analysis of mRNA Stability

To measure the half-life of TRIM58 mRNA and ZBTB2 mRNA, 5 μg/ml of actinomycin D (Sigma-Aldrich) was added into the cell culture medium and total RNA was prepared at the indicated time points and subjected to quantitative RT-PCR analysis using primers.

### Generation of Luciferase Reporter Constructs and Dual-Luciferase Reporter Assay

The full-length 3’UTR of ZBTB2 was generated by PCR and inserted into the pMIR-REPORT Luciferase vector (Promega) just after the stop codon of the reporter gene to generate the wild type (WT) ZBTB2 3’UTR reporter construct. The construct containing deletion of AUF1 potential binding sites was generated using the WT ZBTB2 3’UTR reporter vector by a PCR-based method as recommended in the QuikChange site-directed mutagenesis kit (Agilent Technologies). The promoter fragment containing -1656~+46 sequence of *TRIM58* gene(the transcription start designated as +1)was generated by PCR and inserted into the pGL4.10 Luciferase vector (Promega), a reporter vector without promoter, to construct WT *TRIM58* promoter reporter vector. The vector containing deletion of ZBTB2 potential binding sites was generated using the WT *TRIM58* promoter reporter vector. IHH4 cells were co-transfected with the luciferase reporter vector containing either empty or human ZBTB2 3’UTR. Transfection was performed using Lipofectamine 2000 according to the manufacturer’s instructions (Invitrogen). Firefly and Renila luciferase activities were consecutively measured using the Dual-Luciferase assay as recommended by the Dual-Luciferase assay as recommended by the manufacturer (Promega). The firefly luciferase signal was normalized to the Renila luciferase signal for each individual analysis.

### Chromatin Immunoprecipitation (ChIP)

TPC1 and IHH4 cells were fixed in formaldehydes. The antibody of the target protein was added to form protein-DNA complex. Protein A or Protein G beads to bind the antibody-target protein-DNA complex and precipitate. Remove non-specific binding, and elute to obtain the enriched target protein-DNA complex, de-crosslink, purify the enriched DNA fragment, and perform enrichment analysis.

### RNA Immunoprecipitation (RIP)

To determine interaction of AUF1 with ZBTB2 mRNAs, AUF1 antibody was used to pull down AUF1-interactiong complexes. RNA-binding protein immunoprecipitation kit (Millipore) was used for RIP procedures according to the manufacturer’s protocol. After the antibody was recovered by protein A/G beads, standard quantitative RT-PCR was performed to detect relative mRNA in the precipitates.

### Tissue Specimens and Ethics Statement

A total of 36 PTC tissues and corresponding non-cancerous thyroid tissues were collected from patients who underwent surgical resection at the first affiliated hospital of China Medical University. The diagnosis of PTC was pathologically confirmed either intra- or postoperatively. The peri-tumorous tissues on the same side of the tumor were also collected simultaneously. Those peri-tumorous tissues pathologically diagnosed as normal thyroid tissues were used as paired normal controls. Written informed consent was obtained from all patients and this study was approved by the Ethics Committee of China Medical University and complied with the Declaration of Helsinki. All specimens were frozen in liquid nitrogen immediately and stored at −80°C until use.

### Immunohistochemical Staining

Thyroid tissue specimens were fixed in 4% paraformaldehyde and stored at 4°C. Immunohistochemical staining Thyroid tissue specimens were fixed in 4% paraformaldehyde and stored at 4°C. Antibodies are used to bind specific antigens in thyroid tissue specimens. Thyroid tissue specimens were dehydrated, embedded in paraffin, and sliced. Two percent BSA was blocked for 30 min at room temperature. Incubated with primary and secondary antibodies, SABC complex and DAB chromogenic solution at room temperature for 2 h and 30 min, 30 min and 30 min, respectively. The tissue slices were sealed with gum and took photos for analysis.

### Statistical Analysis

The statistical significance of the difference was analyzed by ANOVA and *post hoc* Dunnett’s test. Statistical significance was defined as P < 0.05. All experiments were repeated at least three times, and data were expressed as the average ± SEM from at least three experiments.

## Result

### AUF1 Knockdown Reduces Proliferation and Invasion of IHH4 Cells

**Figure 1:**
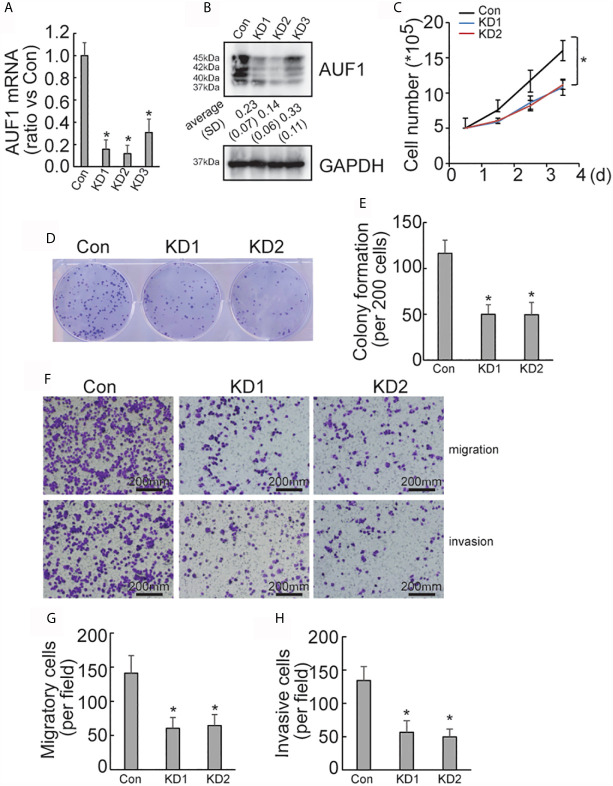
Proliferation, migration, and invasion ability of Con and AUF1-knockdown IHH4 cells. **(A)** The CRISPR/Cas9 system was applied to knock down AUF1 in IHH4 cells, and Real time PCR was performed to clarify knockdown of AUF1 in thyroid cancer cells; **(B)** western blot was performed to verify the knockdown efficiency; **(C)** to evaluate the proliferation ability of IHH4 cells, 1 × 10^5^ cells were seeded and counted on day 1–4 in both the control group (Con) and AUF1-knockdown groups (KD1 and KD2), n = 3, *P < 0.05; **(D)** IHH4 cells in Con, KD1, and KD2 groups were seeded separately as single cells with 200 numbers per well in a six-well plate, and a representative photograph of colony formation was presented; **(E)** the IHH4 colonies were quantitively analyzed by using ImageJ software, n = 3, *P < 0.05; **(F)** Transwell assay containing Matrigel were applied to observe the migration and invasion ability of IHH4 cells in Con, KD1, and KD2 groups, respectively. The representative photographs under microscope (200 μm) were shown; **(G)** the numbers of migrated cells in Transwell assay were counted, n = 3, *P < 0.05; **(H)** the numbers of invading cells in Matrigel containing Transwell assay were counted, n = 3, *P < 0.05.

To explore the effect of AUF1 in human papillary thyroid cancer, AUF1 was knocked down using the CRISPR/Cas9 system in the IHH4 cells. Three gRNAs (KD1, KD2, and KD3) with different sequences were used to target AUF1, and the efficiency of AUF1 knockdown was confirmed by Real time PCR (**Figure 1A**) and Western Blot (**Figure 1B**). KD1 and KD2 were used for the following analyses since more obvious efficiency of knockdown. A cell counting experiment demonstrated that the proliferation rate of IHH4 cells with AUF1 knockdown groups was significantly decreased, when compared with the control cells (**Figure 1C**). In addition, colony formation experiments found that the colony forming abilities were inhibited when endogenous AUF1 was knocked down in IHH4 cells (**Figures 1D, E** **)**. Transwell assay showed that the cells migrated through the membrane to the bottom chamber were significantly lower in AUF1 knockdown cells than the control cells (**Figures 1F, G** **)**. The Matrigel containing Transwell assay demonstrated that the cells passed through the membrane to the bottom chamber were also significantly lower in AUF1 knockdown cells than the control cells (**Figures 1F, H** **)**.

### AUF1 Knockdown Reduces Proliferation and Invasion of TPC1 Cells

**Figure 2:**
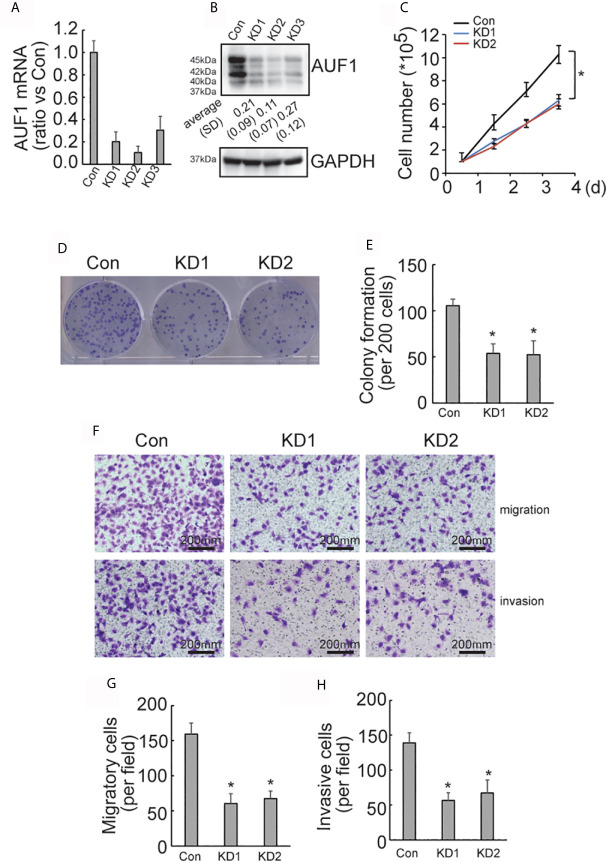
Proliferation, migration, and invasion ability of Con and AUF1-knockdown TPC1 cells. **(A)** The CRISPR/Cas9 system was applied to knock down AUF1 in TPC1 cells, and Real time PCR was performed to clarify knockdown of AUF1 in thyroid cancer cells; **(B)** western blot was performed to verify the knockdown efficiency; **(C)** to evaluate the proliferation ability of TPC1 cells, 1 × 10^5^ cells were seeded and counted on day 1–4 in both the control group (Con) and AUF1-knockdown groups (KD1 and KD2), n = 3, *P < 0.05; **(D)** TPC1 cells in Con, KD1, and KD2 groups were seeded separately as single cells with 200 numbers per well in a six-well plate, and a representative photograph of colony formation was presented; **(E)** the TPC1 colonies were quantitively analyzed by using ImageJ software, n = 3, *P < 0.05; **(F)** Transwell assay containing Matrigel were applied to observe the migration and invasion ability of TPC1 cells in Con, KD1, and KD2 groups, respectively. The representative photographs under microscope (200 μm) were shown; **(G)** the numbers of migrated cells in Transwell assay were counted, n = 3, *P < 0.05; **(H)** the numbers of invading cells in Matrigel containing Transwell assay were counted, n = 3, *P < 0.05.

To further explore the effect of AUF1 in human papillary thyroid cancer, AUF1 was also knocked down in TPC1 cells using the CRISPR/Cas9 system. The efficiency of AUF1 knockdown was confirmed by Real time PCR (**Figure 2A**) and Western Blot (**Figure 2B**). A cell counting experiment showed that the proliferation rate was significantly decreased by AUF1 knockdown in TPC1 cells (**Figure 2C**). A colony formation experiment also demonstrated that the colony forming abilities were inhibited when endogenous AUF1 was knocked down in TPC1 cells (**Figures 2D, E** **)**. Transwell assay observed that the migration ability was significantly suppressed by AUF1 knockdown in TPC1 cells (**Figures 2F, G** **)**. In addition, the Matrigel containing Transwell assay showed that the cells passed through the membrane to the bottom chamber were also significantly decreased by AUF1 knockdown in TPC1 cells (**Figures 2F, H** **)**.

### Upregulation of TRIM58 Is Implicated in AUF1 Knockdown-Mediated Suppression of Proliferation and Invasion of IHH4 and TPC1 Cells

**Figure 3:**
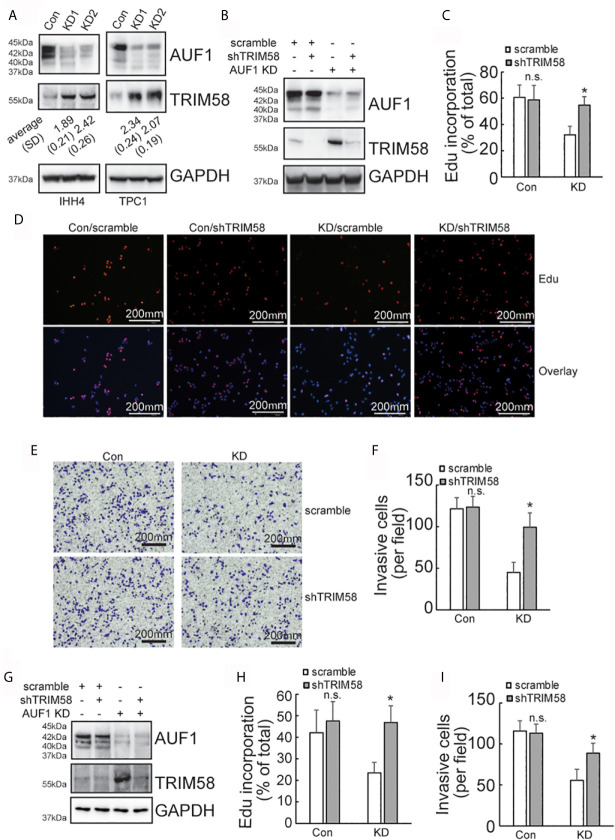
TRIM58 inhibition upregulated the proliferation and invasion ability of AUF1-knockdown papillary thyroid cancer cells. **(A)** Western blot experiments in the corresponding cells with the indicated antibodies; **(B)** Con and KD groups were transfected with scramble or TRIM58 shRNAs in IHH4 cells, and the expression of TRIM58 was measured by western blot; **(C, D)** the proliferation ability of IHH4 cells was assessed using Edu incorporation in scramble-Con and shTRIM58-Con, scramble-KD, and shTRIM58-KD groups (200 μm); **(E, F)** Matrigel containing Transwell assay was applied to analyze the invasion ability of indicated cells and the numbers of invading cells were counted (200 μm); **(G)** Con and KD groups were transfected with scramble or TRIM58 shRNAs in TPC1 cells, and the expression of TRIM58 was measured by western blot; **(H)** the proliferation ability of TPC1 cells was assessed using Edu incorporation; **(I)** Matrigel containing Transwell was used to analyze the invasion ability of indicated TPC1 cells and the numbers of invading cells were analyzed. N.S., not significant; n = 3, *P < 0.05.

SILAC followed by spectrometry screened that 145 proteins and 151 proteins were upregulated and downregulated, respectively, by AUF1 knockdown in IHH4 cells (**Supplementary Data 1**.). *TRIM58* was the gene with the most obvious increase in expression by AUF1 knockdown. Western blot verified that TRIM58 was significantly increased by AUF1 knockdown in both IHH4 and TPC1 cells (**Figure 3A**). To further verify the potential involvement of TRIM58 upregulation in proliferation and invasion ability of human papillary thyroid cancer, specific short hairpin RNAs against TIRM58 (shTRIM58) were applied to interfere TRIM58 expression in Con and AUF1-knockdown IHH4 and TPC1 cells, respectively. The transfection efficiency was verified by western blot (**Figure 3B**). Edu incorporation experiments revealed that TRIM58 knockdown had no significant effects on Con IHH4 cells proliferation, but shTRIM58 group showed significantly higher proliferation ability than the scramble group in AUF1-knockdown group (**Figures 3C, D** **)**. Matrigel containing Transwell assay showed that TRIM58 knockdown had no significant effects on invasion ability of Con, but in KD groups, the number of shTRIM58-KD cells invaded into the chamber bottom was significantly higher than those of scramble-KD cells (**Figures 3E, F** **)**. TRIM58 expression was also knocked down in TPC1 cells (**Figure 3G**). Edu incorporation experiments (**Figure 3H**), and Matrigel contained Transwell assay (**Figure 3I**) showed similar trend as in IHH4 cells.

### AUF1-Knockdown Activates TRIM58 Transcription by Increasing Promoter Activity

**Figure 4:**
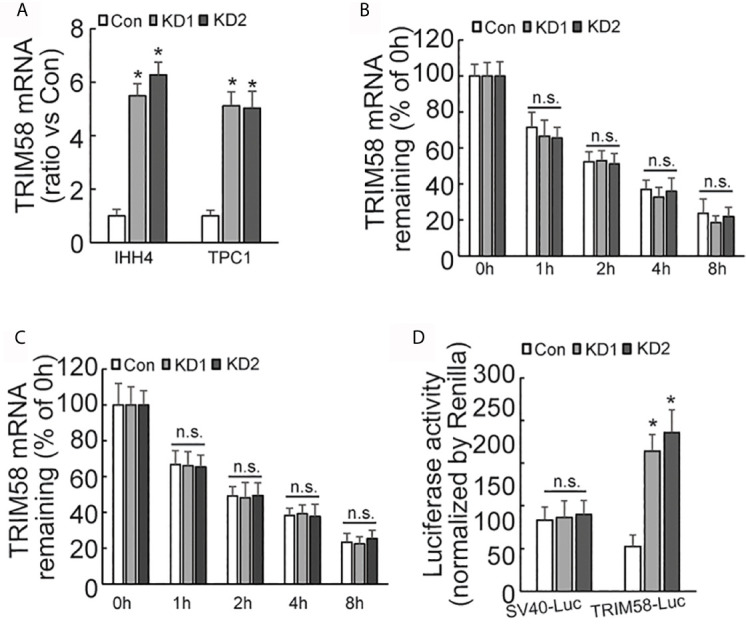
The expression, stability, and promoter activity of TRIM58 mRNA was affected by AUF1 expression in thyroid cancer cells. **(A)** Quantitative RT-PCR was applied to detect the expression level of TRIM58 mRNA of Con, KD1, and KD2 groups in IHH4 and TPC1 cells, and the data was normalized to the corresponding control groups; **(B)** the transcription inhibitor Actinomycin D was applied to Con, KD1, and KD2 groups in IHH4 cells, and the expression level of TRIM58 mRNA decreased as the treatment time prolonged but no significant differences among the control and KD groups were observed; **(C)** the transcription inhibitor Actinomycin D was applied to Con, KD1, and KD2 groups in TPC1 cells, and the expression level of TRIM58 mRNA decreased as the treatment time prolonged but no significant differences among the control and KD groups were observed; **(D)** Luciferase reporter gene vector was constructed for TRIM58 promoter in IHH4 cells for luciferase reporter gene activity detection, and an increased luciferase activity was seen in KD1 and KD2 groups. N.S., not significant; n = 3, *P < 0.05.

Quantitative RT-PCR results showed that the expression of TRIM58 mRNA was significantly increased by AUF1 knockdown (**Figure 4A**). As AUF1 is one of RNA binding proteins (RBPs), which commonly interact with target RNAs and regulate their stability and/or translation. Transcription inhibitor Actinomycin D was then used to inhibit RNA biosynthesis, followed by quantitative RT-PCR to investigate the stability of TRIM58 mRNA. The results showed that the expression level of TRIM58 mRNA decreased as the treatment time prolonged in IHH4 (**Figure 4B**) and TPC1 (**Figure 4C**) cells, but no significant differences of TRIM58 mRNA stability were observed among the control and AUF1 knockdown groups. These data indicated that AUF1 does not affect the expression of TRIM58 at the post-transcription level but at the transcription level. *TRIM58* promoter luciferase reporter gene experiment was further performed in IHH4 cells. It was found that the promoter luciferase activity was significantly increased by AUF1 knockdown (**Figure 4D**), confirming the upregulation of TRIM58 expression at the transcriptional activation level.

### AUF1-Knockdown Activates the Expression of TRIM58 by Increasing the Expression of ZBTB2

**Figure 5:**
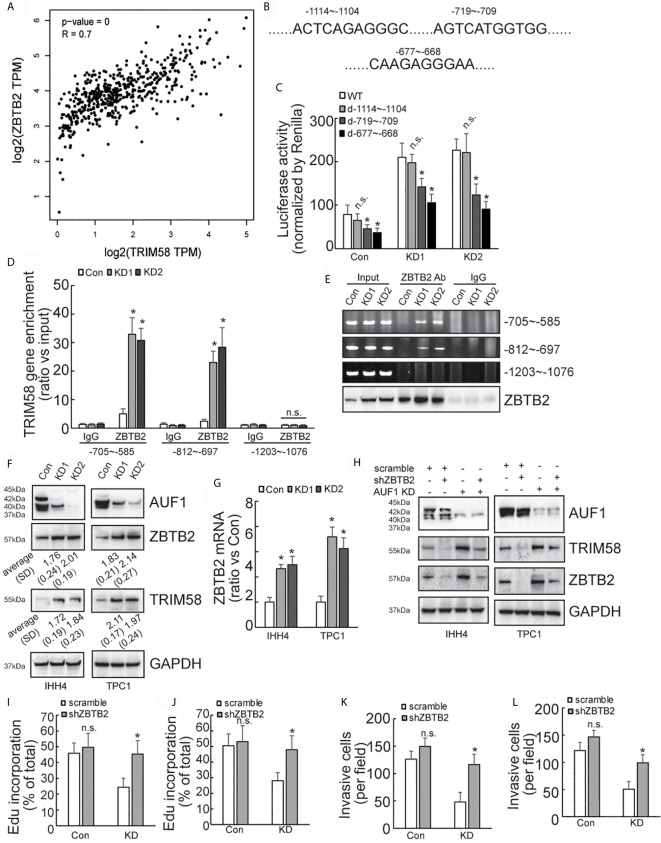
AUF1-knockdown activates the expression of TRIM58 by increasing the expression of ZBTB2. **(A)** TRIM58 and ZBTB2 are co-expressed in thyroid cancer cells, and the two are positively correlated; **(B)** three ZBTB2 potential binding sites on the *TRIM58* promoter; **(C)** the reporter gene activity of the mutants -719~-709 and -677~-668 were reduced in all groups; **(D)** the results of ChIP experiments revealed that AUF1-knockdown increased ZBTB2 enrichment in the segments of -705~-585 and -812~-697 on the *TRIM58* promoters; **(E)** the IP efficiency by ZBTB2 was confirmed by western blot and agarose gel electrophoresis of PCR products of TRIM58 promoter fragments from input as well ChIP was performed. **(F)** AUF1-knockdown increased the expression of ZBTB2 and TRIM58; **(G)** ZBTB2 mRNA expression was evaluated using real time PCR; **(H)** Western blot was performed in IHH4 and TPC1 cells with indicated antibodies, and it was found that shZBTB2 suppressed the expression of TRIM58 in KD groups; **(I)** the proliferation ability of IHH4 cells was assessed using Edu incorporation in scramble-Con and shZBTB2-Con, scramble-KD, and shZBTB2-KD groups; **(J)** the proliferation ability of TPC1 cells was assessed using Edu incorporation; **(K)** Matrigel containing Transwell was used to analyze the invasion ability of indicated IHH4 cells and the numbers of invading cells were analyzed; **(L)** Matrigel containing Transwell was used to analyze the invasion ability of indicated TPC1 cells and the numbers of invading cells were analyzed. N.S., not significant; n = 3, *P < 0.05.

Considering that AUF1 generally regulates gene expression at the post-transcriptional level, we turned to ask whether alternative transcription factor and/or transcription regulatory factor might be implicated in activation of *TRIM58* by AUF1 knockdown in thyroid cancer cells, and ZBTB2 attracted our attention as the following reasons: firstly, ZBTB2 was also included in the upregulated genes by AUF1 knockdown (**Supplementary Data 1**); secondly, bioinformatics analysis showed high co-expression of ZBTB2 and TRIM58 in thyroid cancer (**Figure 5A**); and thirdly, three potential ZBTB2 binding sites were predicted on the *TRIM58* promoter region (**Figure 5B**). Mutant promoter luciferase reporter constructs containing the potential ZBTB2 binding sites were then generated. Luciferase reporter analysis showed that the luciferase activities of two of mutant *TRIM58* promoter luciferase reporter constructs (-719~-709 and -677~-668) were significantly reduced, especially in IHH4 cells with AUF1 knockdown (**Figure 5C**). Furthermore, chromatin immunoprecipitation (ChIP) experiments revealed that ZBTB2 was recruited to the segments of -705~-585 and -812~-697 on the *TRIM58* promoters, which was enhanced by AUF1 knockdown in IHH4 cells (**Figure 5D**). Western blot and agarose gel electrophoresis of PCR products of TRIM58 promoter fragments from input as well ChIP were performed to confirm the IP efficiency by ZBTB2 (**Figure 5E**). Western blot showed that both ZBTB2 and TRIM58 expression was increased by AUF1 knockdown in IHH4 and TPC1 cells (**Figure 5F**). Real time PCR showed that ZBTB2 mRNA expression was evaluated by AUF1 knockdown in IHH4 and TPC1 cells (**Figure 5G**). To explore the potential involvement of ZBTB2 in *TRIM58* transactivation by AUF1 knockdown, ZBTB2 expression was suppressed using shRNAs against ZBTB2 (shZBTB2) in IHH4 and TPC1 cells. Western blot confirmed the efficiency of ZBTB2 suppression by shZBTB2 (**Figure 5H**). Importantly, concurrent with ZBTB2 suppression, TRIM58 expression was significantly decreased, especially in AUF1 knockdown IHH4 and TPC1 cells. ZBTB2 knockdown significantly increased proliferation (**Figures 5I, J**) and invasion (**Figures 5K, L**) of IHH4 (**Figures 5I, K** **)** or TPC1 (**Figures 5J, L** **)** cells with AUF1 knockdown, while had no obvious effects in control cells (**Figures 5I–L**).

### AUF1 Promotes the Degradation of ZBTB2 mRNA by Binding to Its 3’UTR

**Figure 6:**
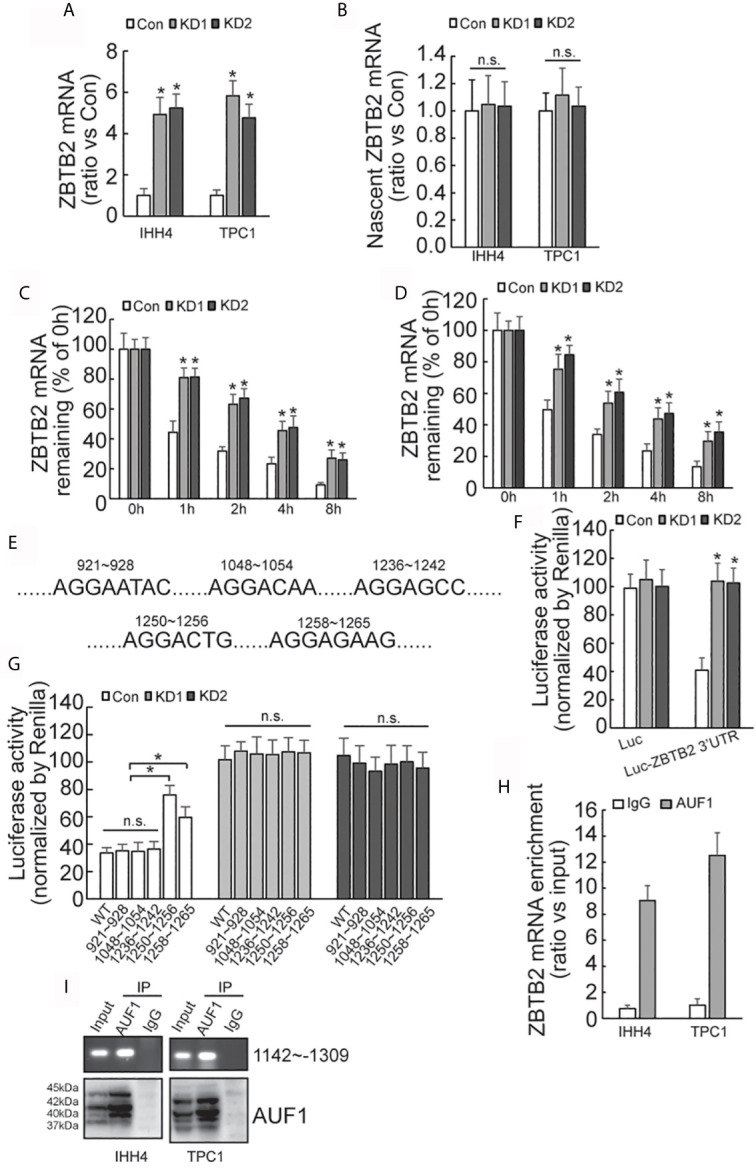
AUF-1 affects ZBTB2 stability by binding to 3’-UTR of ZBTB2 mRNA. **(A)** Quantitative RT-PCR detected the expression level of ZBTB2 mRNA of Con, KD1, and KD2 groups in IHH4 and TPC1 cells; **(B)** qRT-PCR detected the expression of ZBTB2 nascent mRNA of Con, KD1, and KD2 groups in IHH4 and TPC1 cells; **(C, D)** using Actinomycin D to inhibit RNA biosynthesis and explore the stability of ZBTB2 mRNA of Con, KD1, and KD2 groups in IHH4 **(C)** and TPC1 **(D)** cells; **(E)** five potential AUF1 binding sites in the 3’UTR of ZBTB2 mRNA were found *via* bioinformatics; **(F)** an increased luciferase activity was seen in KD1 and KD2 groups after constructing the 3’-UTR of ZBTB2 mRNA into luciferase stop codon; **(G)** mutation of the luciferase reporter gene at in indicated regions and the corresponding luciferase activity was shown; **(H)** the enrichment of AUF1 on ZBTB2 mRNA was confirmed *via* RIP experiment; **(I)** Western blot was used to confirm the IP efficiency by AUF1 antibody and agarose gel electrophoresis of PCR products of ZBTB2 3’UTR was also included. N.S., not significant; n = 3, *P < 0.05.

Quantitative RT-PCR demonstrated that ZBTB2 mRNA level was significantly increased in AUF1-knockdown IHH4 and TPC1 cells (**Figure 6A**). Click-iT Nascent RNA Capture was carried out to capture newly synthesized mRNA, and nascent ZBTB2 mRNA was measured by quantitative RT-PCR. No significant difference of ZBTB2 nascent mRNA expression was observed between the control and AUF1-knockdown cells (**Figure 6B**). RNA biosynthesis was then blocked by addition of Actinomycin D, and it was found that ZBTB2 mRNA stability was significantly increased in IHH4 (**Figure 6C**) or TPC1 (**Figure 6D**) cells with AUF1 knockdown. 3’-untranslated region (3’-UTR) of mRNA plays an important role in post-transcriptional regulation of gene expression (28). Many RNA-binding proteins including AUF1 interact with their binding sites on 3’-UTR to regulate the stability of their target mRNAs (29). Hence, bioinformatics analysis predicted five potential AUF1 binding sites on the 3’-UTR of ZBTB2 transcript (**Figure 6E**). The 3’-UTR of ZBTB2 was inserted just after the stop codon of luciferase reporter, and an increased luciferase activity of the ZBTB2 3’-UTR containing construct was seen in IHH4 cells with AUF1 knockdown (**Figure 6F**). Potential AUF binding site mutant luciferase reporter assay showed an increase of Luciferase activity at segments 1250-1256 and 1258-1265 of ZBTB2 3’-UTR in control, but no significant change of Luciferase activity was found in AUF1 knockdown cells (**Figure 6G**). RNA binding protein immunoprecipitation (RIP) experiment was applied to further confirm the interactions between AUF1 and ZBTB2 mRNA. The results also revealed the enrichment of AUF1 on ZBTB2 mRNAs in both the IHH4 and TPC1 cells (**Figure 6H**). Western blot was performed to confirm the IP efficiency by AUF1 antibody, meanwhile, agarose gel electrophoresis of PCR products of ZBTB2 3’UTR was also performed (**Figure 6I**).

### Correlation of AUF1, ZBTB2, and TRIM58 Expression in Normal and Tumoral Human Thyroid Tissues

**Figure 7:**
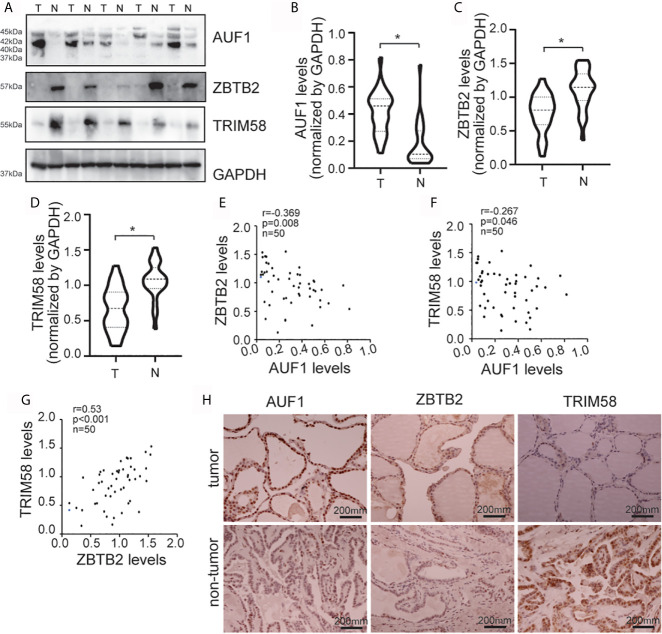
AUF1, ZBTB2, and TRIM58 expression in normal and tumoral human thyroid tissues. **(A)** Western blot was applied to observe the expression difference of AUF1, ZBTB2, and TRIM58 between a total of 36 paired papillary cancer (T) and peripheral normal (N) tissues, a representative blot was shown; **(B)** the Western blot results in panel A were quantitatively analyzed and normalized by GAPDH, and the AUF1 levels in papillary cancer (T) and peripheral normal (N) tissues were counted and compared, n = 36, *P < 0.05; **(C)** and the ZBTB2 levels in papillary cancer (T) and peripheral normal (N) tissues were counted and compared, n = 36, *P < 0.05; **(D)** the TRIM58 levels in papillary cancer (T) and peripheral normal (N) tissues were counted and compared, n = 36, *P < 0.05; **(E)** correlation analysis showed that the protein expression levels of ZBTB2 and AUF1 were negatively correlated. n = 50, p = 0.008, r = −0.369; **(F)** correlation analysis showed that the protein expression levels of TRIM58 and AUF1 were negatively correlated. n = 50, p = 0.046, r = −0.267; **(G)** correlation analysis showed that the protein expression levels of ZBTB2 and TRIM58 were positively correlated. n = 50, p = 0.001, r = 0.53; **(H)** immunohistochemical staining was applied to the human non-tumor tissue and tumor tissue to detect the expression levels of AUF1, ZBTB2, and TRIM58, and the corresponding representative pictures under microscope (scale bar, 200 μm) were shown.

To further study the connections between AUF1, ZBTB2, TRIM58, and thyroid cancer in human, western blot was performed in 36 paired thyroid tissues from papillary cancer (T) and peripheral normal (N) tissues (**Figure 7A**). Results showed that AUF1 expression was increased (**Figure 7B**), while ZBTB2 (**Figure 7C**) and TRIM58 (**Figure 7D**) was reduced in human thyroid tumors, when compared with those in peripheral normal tissues. Quantitative analysis and correlation analysis of the Western Blot results showed that AUF1 was negatively correlated with ZBTB2 (**Figure 7E**) and TRIM58 (**Figure 7F**), whereas ZBTB2 was positively correlated with TRIM58 (**Figure 7G**). Immunohistochemistry confirmed that AUF1 immunohistochemical intensity was increased, while TRIM58 and ZBTB2 immunohistochemical intensities were decreased in papillary cancer tissues (**Figure 7H**).

## Discussion

Rapid and infinite proliferation is the growth characteristics of cancer cells, which can significantly promote the development of the disease. Hence, inhibiting the proliferation of cancer cells can hinder the further deterioration of cancer. Clinical studies revealed the aggressive nature of PTC cells, which causes tumor lesions to spread through the lining of the lymph nodes to the neck lymph nodes and other distant areas. Lesions metastasis is the leading cause of death in cancer patients (30, 31). Therefore, it is essential to investigate the regulatory factors of proliferation, migration, and invasion of PTC cells. The current study demonstrated that knockdown of AUF1 decreased proliferation, migration, and invasion of IHH4 and TPC1 cells. Furthermore, the current study showed that AUF1 was upregulated in most of papillary thyroid cancer tissues, compared with their peripheral normal tissues. Consistent with the current report, it has been reported that AUF1 might be used as a biomarker for thyroid cancer to distinguish tumors malignancy (18). Furthermore, AUF1 knockdown exhibited a decreased cell proliferation in follicle thyroid cancer cells (32). Collectively, these data assigned a tumor-promoting role to AUF1 in thyroid cancer.

TRIM58 is one of the largest families of the E3 ubiquitin connective enzyme, which controls protein stability for almost the entire proteome. It has been reported that decreased TRIM58 expression is associated with a poor patient outcome and promotes invasion of colorectal cancer cells (26). In addition, TRIM58 suppresses the tumor growth of gastric cancer cells by inactivation of b-catenin signaling *via* ubiquitination (33). The current study found that AUF1 knockdown significantly increased TRIM58 expression, and knockdown of TRIM58 partly rescued the reduced proliferation and invasion of IHH4 and TPC1 cells by AUF1 knockdown, indicating that TRIM58 also functions as a tumor suppressive effect in PTC cells. However, the current proteomics data demonstrated that AUF1 knockdown increased TRIM58 expression, simultaneously b-catenin was also increased in thyroid cancer cells. These data indicated that alternative mechanisms other than TRIM58 might be involved in regulation of b-catenin by AUF1 in thyroid cancer cells. The exact mechanisms underlying remain further clarification. As AUF1 is a canonical RBP protein, which regulates gene expression at the post-transcriptional level *via* interacting with 3’UTR of targets. Unexpectedly, the current study demonstrated that AUF1 knockdown activated TRIM58 expression at the transcriptional level. Rather than altering stability of TRIM58 transcript, AUF1 knockdown increased the nascent TRIM58 mRNA level, as well as transcriptional activity of *TRIM58* promoter.

ZBTB2 is a POL family transcription factor. In contrast to function as transcriptional repressor for *p21* and *RelA/p65* genes, the current study demonstrated that ZBTB2 positively regulated transcriptional activation of *TRIM58* gene. Moreover, online database as well as the local data demonstrated the positive coexpression of TRIM58 and ZBTB2 in thyroid cancer tissues. We identified that the mutations on segments -719 ~ -709 and -677 ~ -668 reported a decrease in promoter activity, and more significant in KD groups, which implied that AUF1 may regulate the TRIM58 expression by affecting ZBTB2 binding. Results from the ChIP experiment further confirmed that binding sites of ZBTB2 on TRIM58, as the enrichment of ZBTB2 is significantly higher in segments -705 ~ -585 and -812 ~ -697. Furthermore, in presence of shZBTB2, both the expression of ZBTB2 and TRIM58 were decreased, which implies that AUF1 may affect the expression of TRIM58 by promoting ZBTB2 binding. Therefore, AUF1 may regulate the expression of the TRIM58 by affecting the stability of ZBTB2 mRNA, and then affects the proliferation, migration, and invasion ability of PTC cells.

The current study found that AUF1 knockdown did not affect the expression of ZBTB2 nascent mRNA. Besides, qRT-PCR results revealed that the stability of ZBTB2 mRNA was increased by AUF1 knockdown. These data confirmed that AUF1 affects ZBTB2 expression at the post-transcription level, which is consistent with the image of post-transcriptional gene expression by RBPs. Actually, five potential AUF1 binding sites on 3’-UTR of ZBTB2 were located by using Bioinformatics Surveys analysis, and two binding sites were confirmed by both site-directed mutagenesis and the RIP experiment, which further confirmed the direct regulation of AUF1 on ZBTB2 at the post-transcriptional level in the IHH4 and TPC1 cells.

Overall, our results suggest that AUF1 affecting thyroid cancer cells *via* regulating the expression of ZBTB2 and subsequent TRIM58 expression *in vitro*. Nevertheless, the stability of the expression of these related molecules in the body, including how to be applied in patients after PTC surgery, is still to be studied. Future studies for this purpose may provide new strategies to intervene in the progression and prognosis of thyroid papilloma. However, precision targeted therapy for AUF1 and its downstream ZBTB2 and TRIM58, and its subsequent possibilities in clinical applications, remains to be studied.

## Conclusion

In overall, we suggest that AUF1 may regulate the expression of ZBTB2 by binding to ZBTB2 3’-UTR, affecting the stability of ZBTB2 mRNA, which in turn affects the expression of TRIM58 and regulates the proliferation, migration, and invasion of papillary thyroid cancer cells.

## Data Availability Statement

The original contributions presented in the study are included in the article/**Supplementary Material**. Further inquiries can be directed to the corresponding author.

## Author Contributions

XD, Z-XD, D-LZ, and J-MW contributed to conception and design of the study. TW organized the database. X-YZ and J-YJ performed the statistical analysis. XD wrote the first draft of the manuscript. XD and Z-XD wrote sections of the manuscript. All authors contributed to the article and approved the submitted version.

## Funding

The current work was partly supported by National Natural Science Foundation of China (81470584 and 81902465).

## Conflict of Interest

The authors declare that the research was conducted in the absence of any commercial or financial relationships that could be construed as a potential conflict of interest.
